# Lysine Requirements in Starter‐Phase Japanese Quail (*Coturnix japonica*) Through Dose–Response Modelling: Interactions With Dietary Protein Level

**DOI:** 10.1002/vms3.71142

**Published:** 2026-09-29

**Authors:** Hamid Valizadeh, Mehdi Taghinejad‐Roudbaneh, Ruhollah Kianfar

**Affiliations:** ^1^ Department of Animal Science Faculty of Agriculture and Natural Resources Ta.C. Islamic Azad University Tabriz Iran; ^2^ Department of Animal Science Faculty of Agriculture University of Tabriz Tabriz Iran

**Keywords:** broken‐line regression, dose–response modelling, nitrogen excretion, precision feeding, protein–lysine interaction, starter phase

## Abstract

**Background:**

The diet of Japanese quail (*Coturnix japonica*) during the critical post‐hatching period (0–14 days) provides a basis for long‐term productivity, but the exact lysine requirements are not well characterized in modern high‐yield strains. Precision poultry nutrition requires updated amino acid (AA) recommendations that account for interactions between dietary crude protein (CP) and lysine, the reference AA for ideal protein specification.

**Objectives:**

To determine optimal lysine requirements for growing Japanese quail (0–14 days) at three CP levels (22%, 24%, 26%) using a dose–response method, and to compare linear versus quadratic broken‐line models for estimating lysine thresholds.

**Methods:**

A fully randomized factorial design used 900 Japanese quail chicks allocated to 15 dietary treatments (3 CP × 5 lysine levels: 0.95%, 1.1%, 1.25%, 1.4 %and 1.55 %). Growth performance, feed efficiency and carcass characteristics were evaluated. Lysine requirements were estimated by linear and quadratic broken‐line regression using the non‐linear least squares (NLIN) procedure in Statistical Analysis System (SAS) software version 9.4.

**Results:**

Dietary CP and lysine levels significantly (*p* < 0.01) affected all growth parameters. Optimal lysine (1.4%) maximized body weight (73.93 g), weight gain (66.50 g) and protein efficiency ratio (PER) (2.70). Significant interactions (*p* < 0.05) occurred between CP and lysine for feed intake (FI), feed conversion ratio (FCR) and PER. Lysine requirements for weight gain using the linear broken‐line model were 1.20%, 1.32% and 1.4% for 22%, 24% and 26% CP, respectively, showing superior fit (*R* ^2^ = 66.24%–94.45%) compared to quadratic models.

**Conclusions:**

Lysine requirements of Japanese quail depend on dietary CP level. The linear broken‐line model is more reliable than the quadratic approach. Producers should use 1.32%–1.42% lysine in starter diets, adjusting according to CP content. Future research should confirm these requirements under commercial production conditions and explore interactions with other key AAs in reduced formulae.

## Key Highlights

- Precision lysine requirements for modern Japanese quail strains were established for the critical 0–14‐day starter phase, addressing a key gap in precision poultry nutrition.
- Significant protein × lysine interactions were identified, demonstrating that optimal lysine levels are not static but depend on dietary crude protein content.
- The linear broken‐line model proved superior to the quadratic model for requirement estimation, providing more biologically plausible fits (*R* ^2^ up to 94.45%).
- An optimal lysine level of 1.4% maximized body weight gain and protein efficiency, with a clear performance plateau and decline beyond 1.55%.
- Breast muscle yield was particularly sensitive to lysine supplementation, plateauing at 1.25%–1.4%, which has direct implications for meat‐type quail production.
- The study provides a robust framework for sustainable diet formulation, enabling enhanced growth performance while reducing nitrogen excretion.

## Graphical abstract

In starter Japanese quail, optimal lysine requirements (1.32%–1.42%) depend on dietary crude protein level. Supplementation at 1.4% maximizes body weight gain, protein efficiency, and breast yield, while oversupply (1.55%) impairs performance. Linear broken‐line modelling provides the most reliable requirement estimates for precision feeding.

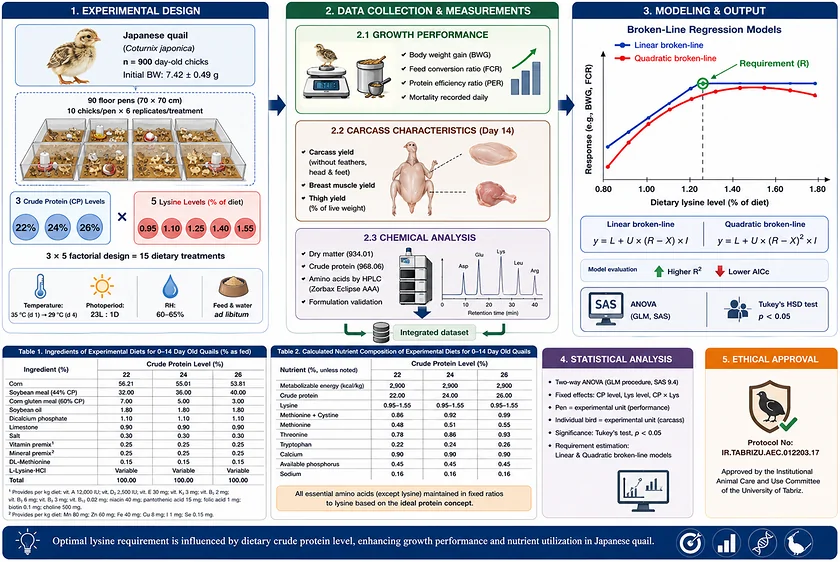

**Abbreviations**
AA: amino acid
AICc: corrected Akaike information criterion
AMEn: apparent metabolizable energy corrected for nitrogen
ANOVA: analysis of variance
BW: body weight
BWG: body weight gain
CI: confidence interval
CP: crude protein
DCAB: dietary cation–anion balance
DCP: dicalcium phosphate
df: degrees of freedom
FCR: feed conversion ratio
FI: feed intake
GLM: general linear model
HPLC: high‐performance liquid chromatography
HSD: honestly significant difference (Tukey's test)
L:D: light:dark (photoperiod)
Lys: lysine
mTOR: mammalian target of rapamycin
NLIN: non‐linear least squares (SAS procedure)
NRC: National Research Council
PER: protein efficiency ratio
SAS: Statistical Analysis System (software)
SE: standard error
SEM: standard error of the mean
TSAA: total sulphur amino acids
Vit & Min Premix: vitamin and mineral premix
WG: weight gain

## 1 Introduction

The poultry diet has gradually moved from a crude protein (CP)‐based approach to a balanced amino acid (AA) approach, with lysine (Lys) becoming the main reference point for ideal protein concepts in poultry species (Knaga et al. 2024). Japanese quail (*Coturnix japonica*) is a high‐value poultry species worldwide, with rapid growth, excellent feed conversion efficiency and early maturation. This makes it an ideal model for nutritional research and sustainable meat production (Alagawany et al. 2014). The initial phase (0–14 days) is a period of intense physiological development and metabolic programming, during which nutritional interventions have a profound effect on lifelong performance (Mehri 2025).

Lys, the second limiting AA in conventional corn–soybean meal poultry diets, serves many important physiological functions beyond protein synthesis (Hasan et al. 2020). As a key component of protein synthesis, Lys directly influences muscle development, growth and total protein synthesis (Alagawany et al. 2020). Recent evidence suggests that Lys also modulates metabolic pathways through its involvement in carnitine synthesis, affecting lipid metabolism and energy homeostasis (Alabi and Adedokun 2025). Lys deficiency impairs growth, reduces feed efficiency and compromises immunity, while oversupply may disturb AA balance and increase nitrogen excretion (Geng et al. 2025).

The concept of ideal protein has revolutionized AA supplementation by defining optimum ratios of essential AAs to Lys. This approach allows more accurate diet formulation aligned with physiological requirements while minimizing nitrogen excretion (Saleh et al. 2021). However, its practical application requires precise determination of Lys requirements under various dietary conditions, particularly across different protein levels used in commercial quail production. Contemporary research suggests that requirements set decades ago may no longer support modern, genetically improved quail strains bred for faster growth and better feed efficiency (Soares et al. 2019).

The interaction between dietary CP content and Lys requirement is a key consideration. Reduced‐protein diets supplemented with crystalline AAs are praised for lowering nitrogen excretion and improving environmental sustainability (Xiao et al. 2023; Wolfe et al. 2024). However, evidence suggests that AA requirements may shift in low‐protein environments, requiring re‐evaluation of established recommendations (Geng et al. 2025; Ullrich et al. 2018).

Previous estimates of Lys requirements for Japanese quail show wide variation (1.20%–1.40% in early growth stages), likely due to methodological differences, genetic variation among quail strains and environmental conditions. Recent factorial modelling estimated maintenance Lys requirement at approximately 156 mg per day per kg^0^·^7^ ^5^ body weight (BW) with an efficiency of 21 mg per g of egg mass in laying quail. However, comparable data for the critical early starter phase remain scarce (Hasanvand et al. 2018; de Carvalho et al. 2023).

Broken‐line regression analysis has been identified as the statistical methodology of choice for nutrient requirement estimation because it effectively identifies inflection points where response rates plateau. Although linear and quadratic broken‐line models are commonly used, their relative suitability for estimating Lys requirements in growing quail has not been sufficiently investigated. The choice of model significantly affects requirement estimates, with practical consequences for feed formulation and production economics (Robbins et al. 2006; Narváez‐Ortiz et al. 2022).

The present study aimed to address existing knowledge gaps by (1) determining optimal Lys requirements in Japanese quail from 0 to 14 days of age at three protein levels (22%, 24% and 26% CP) using a comprehensive dose–response method; (2) assessing the interaction between protein and Lys on growth performance, carcass characteristics and feed efficiency; and (3) comparing the efficacy of linear versus quadratic broken‐line models. The findings provide evidence‐based recommendations to support precision feeding strategies in commercial quail production.

## 2 Materials and Methods

### 2.1 Birds and Housing

In total, 900 Japanese quail (*Coturnix japonica*) chicks (day‐old) weighing 7.42 ± 0.49 g (not kg) were obtained from a commercial hatchery (Codornas VICAMBIA, Brazil). Chicks were randomly allocated to 90 floor pens (70 × 70 cm), each containing 10 chicks, with six replicates per treatment in an environmentally controlled room. The apparatus maintained a 23 L:1 D (light:dark) photoperiod throughout the study, with a gradual decrease in ambient temperature from 35°C on Day old to 29°C by Day 14 according to standard quail handling protocols (Doka et al. 2013; Narváez‐Ortiz et al. 2022). Relative humidity was maintained at 60%–65%. Feed and water were provided ad libitum and adjusted daily.

### 2.2 Experimental Design and Dietary Treatments

The study used a 3 × 5 factorial design with three CP levels (22%, 24% and 26% CP) and five Lys levels (0.95%, 1.1%, 1.25%, 1.4% and 1.55% of diet), totalling 15 dietary treatments. Diets were formulated to meet or exceed National Research Council (NRC) (1994) recommendations for quail, except for protein and Lys which were the variables. The basal diet consisted of corn, soybean meal (44% CP) and corn gluten meal. Protein content was adjusted using soybean meal and crystalline AAs. All essential AAs except Lys were maintained in fixed ratios to Lys according to the ideal protein concept. Based on the recommendations of Rostagno et al. (2017) for Japanese quail, the minimum ratios of essential AAs to Lys were established as 40% for methionine, 68% for total sulphur AAs (TSAA), 67% for threonine, 17% for tryptophan, 108% for arginine, 137% for leucine and 64% for isoleucine, and crystalline AAs were supplemented to ensure these minimum ratios were maintained relative to the Lys content in all experimental diets. Table 1 shows ingredient composition and Table 2 shows calculated nutrient composition.

**TABLE 1:** Ingredients of experimental diets for 0–14‐day‐old quails (% as fed).

Protein level (CP)	22%					24%					26%				
Lysine level (%)	0.95	1.1	1.25	1.4	1.55	0.95	1.1	1.25	1.4	1.55	0.95	1.1	1.25	1.4	1.55
Corn	59.94	59.94	59.94	59.94	59.94	58.43	58.43	58.43	58.43	58.43	54.39	54.39	54.39	54.39	54.39
SBM (44%)	27.74	27.74	27.74	27.74	27.74	27.14	27.14	27.14	27.14	27.14	26.23	26.23	26.23	26.23	26.23
CGM	4.26	4.26	4.26	4.26	4.26	7.20	7.20	7.20	7.20	7.20	10.60	10.60	10.60	10.60	10.60
Canola oil	0.60	0.60	0.60	0.60	0.60	0.46	0.43	0.40	0.37	0.34	0.54	0.54	0.53	0.53	0.52
CS	1.22	1.09	0.95	0.82	0.68	0.00	0.00	0.00	0.00	0.00	0.89	0.80	0.71	0.61	0.52
DCP (17%)	2.04	2.04	2.04	2.04	2.04	2.04	2.04	2.04	2.03	2.03	2.07	2.07	2.07	2.07	2.07
NaHCO_3_	0.13	0.22	0.31	0.40	0.49	0.29	0.34	0.39	0.43	0.48	0.35	0.39	0.44	0.48	0.52
LS	1.23	1.23	1.23	1.23	1.23	1.25	1.25	1.25	1.25	1.25	1.27	1.27	1.27	1.27	1.27
CSalt	0.32	0.26	0.19	0.13	0.06	0.20	0.17	0.14	0.10	0.07	0.17	0.15	0.12	0.10	0.07
VMin premix*	0.50	0.50	0.50	0.50	0.50	0.50	0.50	0.50	0.50	0.50	0.50	0.50	0.50	0.50	0.50
DL‑Met	0.06	0.15	0.24	0.32	0.41	0.03	0.11	0.19	0.27	0.35	0.00	0.08	0.15	0.23	0.30
L‑LysHCl	0.00	0.20	0.39	0.59	0.78	0.00	0.19	0.39	0.58	0.77	0.00	0.19	0.39	0.58	0.77
L‑Thr	0.00	0.09	0.17	0.26	0.34	0.00	0.07	0.15	0.22	0.29	0.00	0.06	0.13	0.19	0.25
L‑Trp	0.00	0.01	0.02	0.03	0.04	0.00	0.01	0.02	0.03	0.04	0.00	0.01	0.02	0.03	0.04
L‑Arg	0.00	0.09	0.18	0.27	0.36	0.00	0.18	0.36	0.54	0.72	0.00	0.08	0.16	0.23	0.31
L‑Val	0.00	0.09	0.18	0.26	0.35	0.00	0.07	0.15	0.22	0.29	0.00	0.06	0.12	0.17	0.23
L‑Glu	1.97	1.48	0.99	0.50	0.01	2.46	1.85	1.23	0.61	0.00	3.00	2.59	2.18	1.76	1.35
L‑Ile	0.00	0.04	0.09	0.13	0.17	0.00	0.03	0.06	0.08	0.11	0.00	0.02	0.03	0.05	0.06
Total	100	100	100	100	100	100	100	100	100	100	100	100	100	100	100

*Note*: Vitamin and mineral premix (Vit & Min Premix) provided per kg of diet: vitamin A (5500 IU), cholecalciferol (1100 IU), vitamin E (11 IU), riboflavin (4.4 mg), Ca pantothenate (12 mg), nicotinic acid (44 mg), choline Cl (220 mg), vitamin B_12_ (6.6 µg), vitamin B_6_ (2.2 mg), menadione (1.1 mg), folic acid (0.55 mg), d‐biotin (0.11 mg), thiamine (1.1 mg), ethoxyquin (125 mg); minerals: Mn (100 mg), Zn (100 mg), Fe (50 mg), Cu (10 mg), I (1 mg).

Abbreviations: CGM, corn gluten meal; CP, crude protein; CS, corn starch; CSalt, common salt; DCP, dicalcium phosphate; DL‐Met, DL‐methionine; L‐Arg, L‐arginine; L‐Glu, L‐glutamic acid; L‐Ile, L‐isoleucine; L‐Lys HCl, L‐lysine hydrochloride; LS, limestone; L‐Thr, L‐threonine; L‐Trp, L‐tryptophan; L‐Val, L‐valine; NaHCO_3_, sodium bicarbonate; SBM, soybean meal (44% CP); VMin, vitamin and mineral.

* Indicates the details of the vitamin and mineral supplement provided.

**TABLE 2:** Calculated nutrient composition of experimental diets for 0–14‐day‐old quails.

CP level				22%				24%					26%			
Lysine	(%)	0.95	1.1	1.25	1.4	1.55	0.95	1.1	1.25	1.4	1.55	0.95	1.1	1.25	1.4	1.55
AMEn	(Mcal/kg)	2.91	2.90	2.90	2.90	2.90	2.92	2.91	2.91	2.90	2.90	2.95	2.95	2.95	2.94	2.94
CP	(%)	22.0	22.0	22.0	22.0	22.0	24.0	24.0	24.0	24.0	24.0	26.0	26.0	26.0	26.0	26.0
LA		1.56	1.56	1.56	1.56	1.56	1.50	1.49	1.48	1.48	1.47	1.42	1.42	1.42	1.42	1.42
CF		2.46	2.46	2.46	2.46	2.46	2.44	2.44	2.44	2.44	2.44	2.36	2.36	2.36	2.36	2.36
Ca		1.01	1.00	1.00	1.00	1.00	1.01	1.01	1.01	1.01	1.01	1.02	1.02	1.02	1.02	1.02
Avail P		0.50	0.50	0.50	0.50	0.50	0.51	0.50	0.50	0.50	0.50	0.51	0.51	0.51	0.51	0.51
K		0.76	0.76	0.76	0.76	0.76	0.75	0.75	0.75	0.75	0.75	0.73	0.73	0.73	0.73	0.73
Cl		0.23	0.23	0.23	0.23	0.23	0.16	0.18	0.20	0.21	0.23	0.14	0.16	0.19	0.21	0.23
Na		0.18	0.18	0.18	0.18	0.18	0.18	0.18	0.18	0.18	0.18	0.18	0.18	0.19	0.19	0.19
Chol	(mg/kg)	1.36	1.36	1.36	1.36	1.36	1.35	1.35	1.35	1.35	1.35	1.31	1.31	1.31	1.31	1.31
Arginine	(%)	1.28	1.36	1.45	1.54	1.63	1.31	1.48	1.66	1.84	2.02	1.33	1.40	1.48	1.55	1.63
Lysine		0.94	1.10	1.25	1.40	1.55	0.95	1.10	1.25	1.40	1.55	0.95	1.10	1.25	1.40	1.55
Met		0.38	0.46	0.55	0.64	0.72	0.38	0.46	0.54	0.62	0.70	0.38	0.46	0.53	0.60	0.67
TSAA		0.71	0.79	0.88	0.97	1.06	0.74	0.82	0.90	0.98	1.05	0.77	0.84	0.91	0.98	1.06
Tyr		0.85	0.85	0.85	0.85	0.85	0.92	0.92	0.92	0.92	0.92	0.99	0.99	0.99	0.99	0.99
Thr		0.76	0.84	0.92	1.00	1.09	0.80	0.87	0.94	1.01	1.09	0.84	0.90	0.97	1.03	1.09
Val		0.94	1.03	1.11	1.20	1.29	1.00	1.07	1.15	1.22	1.29	1.07	1.12	1.18	1.23	1.29
Trp		0.22	0.23	0.24	0.25	0.26	0.22	0.23	0.24	0.25	0.26	0.22	0.23	0.24	0.25	0.27
Leu		1.97	1.97	1.97	1.97	1.97	2.25	2.25	2.25	2.25	2.25	2.54	2.54	2.54	2.54	2.54
Ile		0.84	0.88	0.92	0.97	1.01	0.89	0.92	0.95	0.98	1.01	0.95	0.96	0.98	0.99	1.01
DCAB	(mEq/kg)	265.7	265.7	265.7	265.7	265.7	266.0	265.5	265.0	264.5	264.0	262.4	262.8	263.1	263.6	263.9

Abbreviations: AMEn, apparent metabolizable energy corrected for nitrogen; Avail P, available phosphorus; Ca, calcium; CF, crude fibre; Chol, choline; Cl, chlorine; CP, crude protein; DCAB, dietary cation–anion balance (Na + K − Cl, mEq/kg); Ile, isoleucine; K, potassium; LA, linoleic acid; Leu, leucine; Lys, lysine; Met, methionine; Na, sodium; Thr, threonine; Trp, tryptophan; TSAA, total sulphur amino acids (methionine + cysteine); Tyr, tyrosine; Val, valine.

### 2.3 Data Collection and Measurements

#### 2.3.1 Growth Performance

The individual BW and feed intake (FI) of the pen were recorded weekly. BW gain (BWG) was calculated as the difference between the final and baseline BW. The feed conversion ratio (FCR) was determined as the intake of feed divided by the increase in BW. The protein efficiency ratio (PER) was calculated as the weight gain (WG) divided by the protein intake. Mortality was recorded daily and used to adjust the performance data.

#### 2.3.2 Carcass Characteristics

On Day 14, two birds per replicate (12 birds per treatment) with BW close to the replicate mean were selected. Birds were euthanized by cervical dislocation according to approved animal welfare protocols. Carcass yield (without feathers, head and feet), breast muscle yield and thigh yield were expressed as percentages of live weight.

#### 2.3.3 Chemical Analysis

Feed ingredients and experimental diets were analysed for dry matter (method 934.01), CP (method 968.06) and AA composition by high‐performance liquid chromatography (HPLC) using a Zorbax Eclipse AAA column. Analysed nutrient values confirmed that the experimental diets matched formulations.

### 2.4 Statistical Analysis

Data were analysed using two‑way analysis of variance (ANOVA) with the general linear model (GLM) procedure in Statistical Analysis System (SAS) software (version 9.4; SAS Institute Inc., Cary, North Carolina, USA). The statistical model included dietary CP (22%, 24%, 26%), Lys level (0.95%, 1.1%, 1.25%, 1.4%, 1.55%) and their interaction as fixed effects. Pen served as the experimental unit for growth performance (*n* = 6 replicates per treatment, five birds per pen), while individual bird was the unit for carcass characteristics (*n* = 12 birds per treatment).

Normality of residuals was verified using the Shapiro–Wilk test (*p* > 0.05), and homogeneity of variances was confirmed by Levene's test (*p* > 0.05). When significant differences were detected (*p* < 0.05), treatment means were separated using Tukey's honestly significant difference (HSD) test. Lys requirements were estimated using non‑linear broken‑line regression via the non‐linear least squares (NLIN) procedure in SAS. Two models were fitted:

Linear broken‑line model: *y* = *L* + *U* × (*R* − *X*) × *I* where *y* is the response variable, *X* is the Lys level, *R* is the requirement estimate, *L* is the theoretical maximum response, *U* is the rate constant and *I* = 1 if *X* < *R*, otherwise *I* = 0.

Quadratic broken‑line model: *y* = *L* + *U* × (*R* − *X*)^2^ × *I* where parameters are as described above.

Model fit was evaluated using the coefficient of determination (*R* ^2^) and the corrected Akaike information criterion (AICc). Superior models exhibited higher *R* ^2^ and lower AICc.

## 3 Results

### 3.1 Growth Performance

The main effects of dietary protein and Lys significantly influenced all growth parameters (*p* < 0.01) (Table 3). Increasing CP from 22% to 26% progressively increased final BW (69.30^b^, 69.26^b^ and 71.72^a^ g for 22%, 24% and 26% CP, respectively) and BWG (61.88^b^, 61.83^b^ and 64.29^a^ g). Conversely, FI showed an inverse relationship with protein content: Highest FI occurred at 22% CP (103.41^a^ g per bird) and lowest at 26% CP (101.93^b^ g).

**TABLE 3:** Effects of dietary CP and lysine on growth performance (0–14 days).

Main effects	*n*	BW (g)	BWG (g)	FI (g)	FCR (g/g)	PER
**CP (%)**						
22	30	69.30^b^	61.88^b^	103.41^a^	1.67^c^	2.72^a^
24	30	69.26^b^	61.83^b^	100.99^b^	1.63^b^	2.54^b^
26	30	71.72^a^	64.29^a^	101.93^b^	1.59^a^	2.42^c^
SEM		0.29	0.29	0.52	0.0067	0.010
**Lysine (%)**						
0.95	18	64.67^e^	57.24^e^	100.77^b^	1.76^d^	2.37^d^
1.1	18	68.11^d^	60.68^d^	102.50^a^ ^b^	1.68^c^	2.47^c^
1.25	18	70.99^c^	63.56^c^	100.75^b^	1.58^a^	2.63^b^
1.4	18	73.93^a^	66.50^a^	102.83^a^	1.54^b^	2.70^a^
1.55	18	72.76^b^	65.34^b^	103.71^a^	1.58^a^	2.63^b^
SEM		0.37	0.37	0.67	0.0075	0.014
**ANOVA**	**df**	**Probabilities**				
Lysine	4	<0.01	<0.01	0.01	<0.01	<0.01
CP	2	<0.01	<0.01	<0.01	<0.01	<0.01
Lys × CP	8	0.10	0.10	0.01	<0.01	<0.01
Error	73					
*R* ^2^		85.9	86.7	40.8	85.7	91.8

*Note*: Each mean represents six replicate pens with five birds per pen (30 birds per treatment for main effects of CP, and 18 pens per lysine level). Different letters within each main effect differ significantly (*p* < 0.05).

Abbreviations:  BW, body weight; BWG, body weight gain; df, degrees of freedom; FCR, feed conversion ratio (FI/BWG); FI, feed intake; *n*, number of replicates; PER, protein efficiency ratio (BWG / protein intake); SEM, standard error of the mean.

Lys supplementation produced a quadratic response. Optimal BW (73.93^a^ g), BWG (66.50^a^ g) and PER (2.70^a^) were achieved at 1.4% dietary Lys. Further increase to 1.55% Lys reduced performance in all these parameters, indicating an optimal threshold beyond which additional Lys is not beneficial. Significant protein × Lys interactions (*p* < 0.05) were observed for FI, FCR and PER. At lower protein (22% CP), performance improved with increasing Lys up to 1.4%, whereas at higher protein levels (24% and 26% CP), the response was less pronounced with little improvement beyond 1.25% Lys.

### 3.2 Carcass Characteristics

Carcass parameters were significantly affected by dietary treatments (Table 4). Carcass yield showed a quadratic response to increasing protein levels, peaking at 24% CP (72.2%) before declining at 26% CP (71.9%). Breast muscle yield followed a similar pattern, with the highest value (32.3%) at 24% CP. Thigh muscle yield showed minimal variation across protein levels.

**TABLE 4:** Effects of dietary CP and lysine on carcass characteristics.

Main effects	*n*	PER	Carcass weight (g)	Carcass yield (%)	Breast weight (g)	Breast yield (%)	Thigh weight (g)	Thigh yield (%)
**CP (%)**								
22	30	2.72^a^	49.3^b^	71.8	20.8^c^	29.0^c^	13.5^b^	19.8^a^
24	30	2.54^b^	51.7^a^	72.2	22.1^b^	32.3^a^	14.2^a^	19.7^a^
26	30	2.42^c^	52.2^a^	71.9	22.7^a^	31.2^b^	13.8^a^ ^b^	19.1^b^
SEM		0.010	0.43	0.28	0.18	0.18	0.13	0.11
**Lysine (%)**								
0.95	18	2.37^d^	46.8^c^	71.8	19.3^c^	29.6^c^	12.5^c^	19.2^c^
1.1	18	2.47^c^	50.8^b^	72.4	21.2^b^	30.2^c^	13.5^b^	19.2^c^
1.25	18	2.63^b^	51.4^b^	71.8	22.9^a^	32.0^a^	14.4^a^	20.1^a^
1.4	18	2.70^a^	53.2^a^	71.8	23.0^a^	31.2^b^	14.5^a^	19.7^b^
1.55	18	2.63^b^	53.3^a^	72.2	23.0^a^	31.2^b^	14.4^a^	19.6^b^ ^c^
SEM		0.014	0.56	0.36	0.23	0.23	0.17	0.14
**ANOVA**	**df**	**Probabilities**						
Lysine	4	<0.01	<0.01	0.53	<0.01	<0.01	<0.01	<0.01
CP	2	<0.01	<0.01	0.59	<0.01	<0.01	<0.01	<0.01
Lys × CP	8	<0.01	0.20	0.83	0.99	0.03	0.48	0.49
Error	73							
*R* ^2^		91.8	62.9	13.5	77.9	78.4	62.5	47.6

*Note*: Different letters in each column indicate significant differences (*p* < 0.05). PER = weight gain (g) / protein fed (g). *n* is the number of replicates. Each mean represents six replicate pens with five birds per pen (30 birds per treatment for main effects of CP, and 18 pens per lysine level).

Lys supplementation linearly increased carcass weight up to 1.4% (53.2^a^ g) without further improvement at 1.55%. Breast muscle yield was particularly sensitive to Lys levels, increasing from 29.6%^c^ at 0.95% Lys to 32.0%^a^ at 1.25% Lys, then stabilizing. A significant protein × Lys interaction (*p*  =  0.03) for breast yield indicated that the optimal Lys level for breast development varies with dietary protein content: Higher protein diets required relatively less additional Lys to reach maximum breast yield.

### 3.3 Lys Requirement Estimates

The estimated Lys requirements using a broken‐line regression analysis showed significant differences depending on response criteria, protein content and statistical model used (Tables 5 and 6). Linear broken‐line models consistently provided better goodness of fit (*R* ^2^: 66.24–94.45) than the quadratic approach (*R* ^2^: 53.20–93.67) for most of the response criteria.

**TABLE 5:** Optimal lysine requirements (linear broken‐line model).

CP (%)	Response	Equation	Requirement (%)	95% CI	SE	*R* ^2^
22	WG	*y* = 64.41 − 35.67 (1.20 − *x*)	1.20	1.10–1.28	0.046	74.91
24		*y* = 64.83 − 23.22 (1.32 − *x*)	1.32	1.28–1.36	0.021	94.45
26		*y* = 67.80 − 19.31 (1.40 − *x*)	1.40	1.29–1.54	0.072	75.80
22	BW	*y* = 72.44 − 24.67 (1.32 − *x*)	1.32	1.22–1.39	0.044	77.64
24		*y* = 71.72 − 23.22 (1.31 − *x*)	1.31	1.26–1.35	0.022	93.06
26		*y* = 75.06 − 18.69 (1.40 − *x*)	1.40	1.26–1.53	0.069	77.14
22	FCR	*y* = 1.61 + 0.72 (1.25 − *x*)	1.25	1.08–1.41	0.083	67.18
24		*y* = 1.58 + 0.50 (1.30 − *x*)	1.30	1.25–1.35	0.026	89.92
26		*y* = 1.50 + 0.55 (1.33 − *x*)	1.33	1.26–1.41	0.037	86.25
22	PER	*y* = 2.82 − 1.12 (1.25 − *x*)	1.25	1.07–1.41	0.087	65.08
24		*y* = 2.44 − 0.74 (1.31 − *x*)	1.31	1.26–1.36	0.027	90.43
26		*y* = 2.55 − 0.81 (1.34 − *x*)	1.34	1.26–1.46	0.041	85.57
22	Breast Wt	*y* = 21.94 − 12.49 (1.22 − *x*)	1.22	1.11–1.34	0.060	73.61
24		*y* = 23.33 − 13.75 (1.23 − *x*)	1.23	1.12–1.33	0.055	74.99
26		*y* = 23.97 − 11.49 (1.27 − *x*)	1.27	1.19–1.35	0.043	70.75
22	Thigh Wt	*y* = 14.86 − 6.96 (1.23 − *x*)	1.23	1.07–1.40	0.087	61.21
24		*y* = 14.18 − 6.48 (1.25 − *x*)	1.25	1.04–1.45	0.10	56.99
26		*y* = 14.72 − 4.41 (1.40 − *x*)	1.40	1.17–1.54	0.11	66.24

*Note*: Linear broken‐line model form: *y* = *a* − *b* × (*R* − *x*) for *x* ≤ *R*, and *y* = *a* for *x* > *R*, where *R* is the breakpoint (requirement). Breast Wt (g), thigh Wt (g).

Abbreviations: BW, final body weight (g); CI, confidence interval; CP, crude protein; FCR, feed conversion ratio; PER, protein efficiency ratio; SE, standard error of the breakpoint; WG, body weight gain (g).

**TABLE 6:** Optimal lysine requirements (quadratic broken‐line model).

CP (%)	Response	Equation	Requirement (%)	95% CI	SE	*R* ^2^
22	WG	*y* = 64.91 − 41.72 (1.42 − *x*)^2^	1.42	1.24–1.60	0.091	77.25
24		*y* = 65.21 − 23.92 (1.46 − *x*)^2^	1.46	1.44–1.68	0.061	93.67
26		*y* = 65.21 − 23.92 (1.52 − *x*)^2^	1.52	1.20–1.65	0.216	70.45
22	BW	*y* = 72.31 − 39.13 (1.43 − *x*)^2^	1.43	1.25–1.61	0.092	78.44
24		*y* = 71.71 − 37.14 (1.49 − *x*)^2^	1.49	1.40–1.65	0.063	91.53
26		*y* = 75.61 − 16.94 (1.54 − *x*)^2^	1.54	1.17–2.19	0.260	72.35
22	FCR	*y* = 1.61 + 1.33 (1.35 − *x*)^2^	1.35	1.15–1.56	0.104	65.42
24		*y* = 1.57 + 0.66 (1.48 − *x*)^2^	1.48	1.35–1.60	0.064	90.30
26		*y* = 1.50 + 0.58 (1.50 − *x*)^2^	1.50	1.36–1.77	0.106	83.57
22	PER	*y* = 2.82 − 1.98 (1.36 − *x*)^2^	1.36	1.14–1.58	0.152	63.01
24		*y* = 2.64 − 0.90 (1.50 − *x*)^2^	1.50	1.38–1.63	0.065	90.58
26		*y* = 2.56 − 0.81 (1.56 − *x*)^2^	1.56	1.34–1.73	0.125	82.80
22	Breast Wt	*y* = 21.98 − 21.13 (1.36 − *x*)^2^	1.36	1.18–1.53	0.089	72.85
24		*y* = 23.42 − 21.42 (1.38 − *x*)^2^	1.38	1.25–1.61	0.090	74.55
26		*y* = 23.99 − 17.58 (1.41 − *x*)^2^	1.41	1.20–1.62	0.116	70.13
22	Thigh Wt	*y* = 14.87 − 12.79 (1.35 − *x*)^2^	1.35	1.12–1.58	0.110	60.00
24		*y* = 14.15 − 13.99 (1.32 − *x*)^2^	1.32	1.08–1.57	0.120	54.78
26		*y* = 14.56 − 6.40 (1.51 − *x*)^2^	1.51	1.13–1.88	0.190	53.20

*Note*: Quadratic broken‐line model form: *y* = *a* − *b* × (*R* − *x*) for *x* ≤ *R*, and *y* = *a* for *x* > *R*, where *R* is the breakpoint (requirement). Breast Wt (g), thigh Wt (g).

Abbreviations: BW, final body weight (g); CI, confidence interval; CP, crude protein; FCR, feed conversion ratio; PER, protein efficiency ratio; SE, standard error of the breakpoint; WG, body weight gain (g).

In the case of WG, Lys requirements estimated from a linear model ranged from 1.32 to 1.42 mg, with a lowest requirement at 24 mg CP and a higher requirement at 22 and 26 mg CP. On the other hand, requirements for FCR optimization showed a greater variability (1.25–1.48) with the highest requirement at 24% of CP. The quadratic model consistently produced higher estimates of requirements than the linear model for all criteria and protein levels, with differences ranging from 0.04 to 0.07 percentage points.

## 4 Discussion

### 4.1 Growth Performance Responses

The significant increase in BWG and FCR efficiency with increasing CP from 22% to 26% aligns with the AA demands of modern, fast‐growing quail strains during the intensive starter phase. These findings confirm recent research suggesting that historical protein recommendations may underestimate the needs of continuously improved quail lines. The inverse relationship between protein content and FI suggests that increased nutrient density and satiety signals associated with higher protein intake may be mediated by improved AA balance and metabolic efficiency (Awad et al. 2014; Wen et al. 2016).

The quadratic response of growth parameters to increasing Lys reflects classic dose–response dynamics. The performance plateau above 1.4% Lys indicates a physiological threshold beyond which protein synthesis becomes limited by other AAs. The subsequent decline at 1.55% Lys suggests possible AA imbalance, perhaps due to disruption of neutral AA transport or increased metabolic costs from AA over‐catabolism and nitrogen excretion (Bertocchi et al. 2019; Mousa et al. 2023).

### 4.2 Protein × Lys Interactions

Significant protein × Lys interactions for feed efficiency parameters support the hypothesis that Lys requirement is not static but context‐dependent. The stronger Lys response at lower protein (22% CP) indicates greater reliance on supplemental Lys when total protein is limited, consistent with complementary AA action. Conversely, the attenuated response at higher protein (26% CP) suggests that native protein sources supply sufficient Lys with less need for supplementation (Hurwitz et al. 1998; Duncan et al. 1996).

These interactions have practical implications for feed formulation. In reduced‐protein diets, precise Lys supplementation is critical to maintain performance, whereas in high‐protein diets, the margin for error is larger. This may explain the variable Lys requirement estimates in the literature, as different studies used different basal protein levels. Our findings reinforce the need to consider the full dietary context when setting AA recommendations (Songuine et al. 2024; Chrystal et al. 2020).

### 4.3 Carcass Composition Modifications

The quadratic response of carcass and breast muscle yields to increasing protein, with an optimum at 24% CP, indicates that protein utilization efficiency declines above a certain threshold. This pattern may reflect metabolic inefficiencies of high protein intake, including increased heat production and liver workload from nitrogen deamination and urea synthesis. The superior breast muscle development at moderate protein (24%) with adequate Lys emphasizes the importance of balanced AA intake rather than maximal protein content for meat yield (Konieczka et al. 2024).

The particular sensitivity of breast muscle to Lys supplementation is consistent with the high Lys content of muscle proteins and Lys's central role as a limiting AA for protein synthesis. The plateau in breast yield above 1.25%–1.4% Lys indicates that skeletal muscle development is limited by other nutritional or genetic factors once Lys requirement is met. This finding is especially important for meat‐type quail production, where breast meat yield is a key economic driver (Suen et al. 2025; Holeček 2025).

### 4.4 Modelling Approaches for Requirement Estimation

The superior performance of the linear broken‐line model for Lys requirement estimation aligns with previous poultry nutrition research. The linear approach better captures the biological reality of AA utilization: response increases linearly until the requirement is reached, then plateaus as other nutrients become limiting. The consistently higher quadratic model estimates likely represent overfitting, especially given the pronounced peaks observed in our data (Ramirez‐Camba and Levesque 2023; Taghinejad et al. 2011; Nogueira et al. 2021).

Variation in requirement estimates by response criterion underscores the context‐dependent nature of optimal Lys levels. The higher requirement for WG versus FCR at 22% CP (1.42% vs. 1.25%) suggests that maximizing growth potential requires a more generous Lys supply than just efficient feed use. This distinction is practically important for producers targeting specific outcomes. The factorial approach combining maintenance and growth requirements provides a physiological framework for understanding these differences across performance metrics (Fatan et al. 2023; Ghorab et al. 2025; Tsuchino et al. 2024).

### 4.5 Comparative Analysis With Existing Literature

Our Lys requirements (1.32%–1.42% for BWG) are generally higher than historical recommendations but align with recent studies using modern quail strains and advanced statistical methods. For example, a 2023 study estimated Lys requirements of Japanese quail at 284 mg/day (approximately 1.35%–1.45% of diet) using a monomolecular factorial model. Similarly, research in laying hens reported maintenance requirements of 156 mg per kg^0^·^7^ ^5^ BW per day and utilization efficiency of 21 mg per g egg mass (Siqueira et al. 2021; Lima et al. 2016).

The higher requirements in our study are likely due to both genetic progress in growth potential and methodological differences in requirement estimation. Our findings confirm the evolving nature of nutrient recommendations for continuously improving animal populations, necessitating regular re‐evaluation of established standards. The requirements we establish specifically address the needs of modern meat‐type quail strains during the critical starter phase, filling a significant gap in precision quail feeding (Siqueira et al. 2021; Ghorab et al. 2025; de Carvalho et al. 2023).

### 4.6 Mechanistic Explanations for Observed Responses

Physiological responses to Lys supplementation can be interpreted through established biochemical pathways regulating protein metabolism. As the first limiting AA in many poultry feedstuffs, Lys controls the rate of protein synthesis directly via mammalian target of rapamycin (*mTOR*) pathway activation and indirectly through its role in carnitine synthesis, which influences fatty acid transport into mitochondria for energy production. Suboptimal Lys intake inhibits mTOR activation, reducing translational efficiency and cell growth rate (Hasanvand et al. 2018; Nogueira et al. 2021; Liao et al. 2025). This mechanism explains the rapid performance improvement with increasing Lys up to the required threshold.

Metabolic costs associated with Lys catabolism also contribute to the observed response patterns. Excessive Lys intake requires deamination and urea synthesis, processes that generate metabolic heat and increase liver and kidney workload. This metabolic penalty manifests as reduced feed efficiency and protein utilization at supramaximal Lys levels, as demonstrated by the lower PER at 1.55% Lys in our study (Galili et al. 2001; Xue et al. 2011).

### 4.7 Study Limitations

Although this study provides comprehensive Lys requirement estimates at multiple protein levels, several limitations should be recognized. First, the experimental conditions used optimal environmental controls that may not fully represent commercial production environments with associated stresses and disease challenges. Validation under field conditions would enhance practical application. Second, the study focused exclusively on the starter phase (0–14 days), whereas full production cycles require understanding of nutrient requirements at all growth stages. Future research should determine Lys requirements for subsequent growth phases to enable whole‐life feeding strategies.

Third, economic aspects of Lys supplementation were not explicitly addressed. Feed formulation decisions must balance biological response with economic optimization, which may shift requirement recommendations depending on ingredient costs. Future studies including economic analysis would increase practical usefulness. Finally, interactions between Lys and other key AAs, especially in reduced‐protein diets, warrant further investigation. Although we maintained ideal protein ratios, possible synergistic or antagonistic interactions under practical feeding conditions merit additional study.

### 4.8 Recommendations and Future Perspectives

Based on current findings, Japanese quail producers should provide Lys levels in the starter phase (0–14 days) between 1.32% and 1.42% of diet, adjusting according to dietary protein content. For 22% and 26% CP diets, the upper end of the range (1.40%–1.42%) is recommended, whereas 1.32%–1.35% Lys is adequate for 24% CP diets. These recommendations prioritize both growth performance and feed efficiency while minimizing nitrogen excretion.

Future research should explore several emerging aspects: First, developing dynamic requirement models accounting for time‐related changes in Lys needs along the growth curve; second, testing Lys interactions with alternative protein sources and feed additives; third, investigating environmental factors (especially thermal stress) on Lys metabolism and requirements in the context of climate change and integrating omics technologies (transcriptomics, proteomics, metabolomics) into quail nutrition research to identify biomarkers for AA status.

## 5 Summary and Conclusion

This comprehensive study shows that Lys requirements for growing Japanese quail during the starter phase (0–14 days) range from 1.32% to 1.42% of diet depending on protein content and performance targets. The linear broken‐line model provided superior requirement estimates compared to the quadratic approach, yielding more biologically plausible values with better goodness of fit. Significant protein × Lys interactions highlight the importance of dietary context in AA recommendations. Optimal Lys level of 1.4% resulted in maximum BW, WG and protein efficiency, while the higher level (1.55%) decreased performance, indicating negative effects of over supplementation. Breast muscle yield was particularly sensitive to Lys, plateauing at 1.25%–1.4% depending on protein content. Producers should adjust Lys according to CP: 1.40%–1.42% for 22% or 26% CP diets, and 1.32%–1.35% for 24% CP diets. Future research should validate these findings under commercial conditions and explore interactions with other AAs in reduced‐protein formulations.

## Author Contributions

**Hamid Valizadeh**: investigation, methodology, project administration, funding acquisition. **Mehdi Taghinejad‐Roudbaneh**: supervision, writing – review and editing. **Ruhollah Kianfar**: data curation, formal analysis, writing – original draft.

## Funding

The authors have nothing to report.

## Ethics Statement

All experimental procedures were reviewed and approved by the Institutional Animal Care and Use Committee of the University of Tabriz (Protocol No: IR.TABRIZU.AEC.012203.17; date of approval: 14 March 2023) and carried out in accordance with established guidelines for the ethical treatment of research animals.

## Conflicts of Interest

The authors declare no conflicts of interest.

## Data Availability

The data presented in this study are available on request from the corresponding author. The data are not publicly available due to privacy restrictions.
